# MicroRNA-3145 as a potential therapeutic target for hepatitis B virus: inhibition of viral replication via downregulation of HBS and HBX

**DOI:** 10.3389/fmicb.2024.1499216

**Published:** 2025-01-06

**Authors:** Amrizal Muchtar, Daichi Onomura, Dan Ding, Hironori Nishitsuji, Kunitada Shimotohno, Shunpei Okada, Keiji Ueda, Koichi Watashi, Takaji Wakita, Kei Iida, Hironori Yoshiyama, Hisashi Iizasa

**Affiliations:** ^1^Department of Microbiology, Faculty of Medicine, Shimane University, Izumo, Japan; ^2^Faculty of Medicine, Universitas Muslim Indonesia, Makassar, Indonesia; ^3^Division of Virology, Department of Infection and Immunity, Jichi Medical University, Shimotsuke, Japan; ^4^Department of Pathology, Changhai Hospital, Naval Medical University, Shanghai, China; ^5^Department of Virology and Parasitology, Fujita Health University School of Medicine, Toyoake, Japan; ^6^Genome Medical Sciences Project, National Center for Global Health and Medicine, Ichikawa, Japan; ^7^Division of Virology, Department of Microbiology and Immunology, Osaka University Graduate School of Medicine, Suita, Japan; ^8^Department of Virology II, National Institute of Infectious Diseases, Tokyo, Japan; ^9^Faculty of Science and Engineering, Kindai University, Higashiōsaka, Japan; ^10^Center for Cancer Immunotherapy and Immunobiology, Graduate School of Medicine, Kyoto University, Kyoto, Japan

**Keywords:** hepatitis B virus, microRNA, endoplasmic reticulum stress, anti-viral drug, hepatocellular carcinoma

## Abstract

Current treatments for hepatitis B virus (HBV), such as interferons and nucleic acid analogs, have limitations due to side effects like depression and the development of drug-resistant mutants, highlighting the need for new therapeutic approaches. In this study, we identified microRNA-3145 (miR-3145) as a host-derived miRNA with antiviral activity that is upregulated in primary hepatocytes during HBV infection. The expression of its precursor, pri-miR-3145, increased in response to the the virus infection, and miR-3145 downregulated the hepatitis B virus S (HBS) antigen and hepatitis B virus X (HBX), thereby inhibiting viral replication. The binding site for miR-3145 was located in the HBV polymerase (pol) region, as experimentally confirmed. Moreover, overexpression of HBS and HBX induced pri-miR-3145 expression through endoplasmic reticulum stress. The expression of pri-miR-3145 showed a strong correlation with the Nance–Horan syndrome-like 1 (*NHSL1*) gene, as it is encoded within an intron of NHSL1, and higher NHSL1 expression in hepatocellular carcinoma patients with HBV infection was associated with better prognosis. These findings suggest that miR-3145-3p, along with small molecules targeting its binding sites, holds promise as a potential therapeutic candidate for HBV treatment.

## Introduction

Hepatitis B virus (HBV) infection is a major cause of chronic hepatitis and hepatocellular carcinoma (HCC), leading to significant global health challenges (Yuen, 2021; Manns and Maasoumy, 2022). While a vaccine against HBV exists, the virus continues to affect millions, with approximately 257 million people living with chronic HBV and 887,000 deaths in 2015 alone (Yuen, 2021). Effective antiviral treatments are essential for preventing and managing HBV-related HCC. Currently, nucleos(t)ide reverse-transcriptase inhibitors (NRTIs) and polyethylene glycol-modified interferons (PEG-IFNs) are the primary treatments used to reduce HBV viral load and slow the progression of liver complications, such as fibrosis, cirrhosis, and HCC (Clark and Hu, 2015). However, long-term use of NRTIs often results in drug-resistant HBV strains (Kim et al., 2014). Additionally, PEG-IFN therapy has a limited success rate, with only 37–60% of patients achieving HBS antigen clearance, and some patients remain unresponsive (Wong et al., 2010). Moreover, PEG-IFN treatment can cause adverse effects, including depression (Tang et al., 2018). Consequently, there is a critical need for new antiviral agents beyond NRTIs and PEG-IFNs.

HBV replication involves the expression of several viral RNAs, including pregenomic RNA (pgRNA), pre-core mRNA (pcRNA), preS1 mRNA, preS2/S mRNA, and HBV X (HBX) mRNA, which are transcribed from multiple promoters within the viral genome (Yuen, 2021). The viral genomic DNA is synthesized through reverse transcription, using pgRNA as a template. Since pgRNA is localized in the cytoplasm prior to being encapsulated in viral core proteins, it is vulnerable to degradation by host microRNAs (miRNAs) (Sarfaraz et al., 2022). This natural degradation process can be harnessed for antiviral therapy.

miRNAs are small, non-coding RNA molecules, typically 22–25 nucleotides long, that regulate gene expression by binding to complementary sequences in the 3′-untranslated regions (3′-UTRs) of target mRNAs. This interaction leads to mRNA degradation or translational repression, mediated by the RNA-induced silencing complex (RISC) (Bartel, 2009). The biogenesis of miRNAs begins with the processing of primary miRNAs (pri-miRNAs) in the nucleus, which are cleaved into precursor miRNAs (pre-miRNAs) by Drosha, and subsequently processed in the cytoplasm by Dicer into mature miRNA duplexes. Some host-derived miRNAs, such as miR-122 and miR-199, have shown anti-HBV properties (Lamontagne et al., 2015; Liu et al., 2019; Naito et al., 2018), but their therapeutic potential is limited due to poor sequence conservation among HBV strains (Hayer et al., 2013) and reduced expression levels in HBV-infected cells (Sartorius et al., 2019; Wakasugi et al., 2018). Notably, miR-302c-3p has demonstrated significant anti-HBV effects in animal studies (Hamada-Tsutsumi et al., 2019).

In this study, we identified miR-3145, a miRNA encoded within the intronic region of the Nance–Horan syndrome-like 1 (*NHSL1*) gene, as a potential antiviral agent against HBV. Higher expression of *NHSL1* in HCC patients is associated with a favorable prognosis. Furthermore, antiviral factor nuclear factor 90 (NF90) is known to promote miR-3145 production (Grasso et al., 2020), and miR-3145 has previously been shown to inhibit the influenza virus by reducing viral polymerase 1 expression (Khongnomnan et al., 2015). Our findings reveal that the expression of both pri-miR-3145 and mature miR-3145 is upregulated in response to HBV infection. Moreover, miR-3145 exhibits antiviral activity by targeting the highly conserved *pol* region of the HBV genome.

Endoplasmic reticulum (ER) stress plays a significant role in the response to HBV infection. Binding immunoglobulin proteins (BiPs), located on the ER membrane, normally inhibit stress sensors like activated transcription factor 6 (ATF6). However, when misfolded proteins accumulate in the ER, BiPs bind to these excess proteins, leading to their degradation via the proteasome. This dissociation of BiPs from stress sensors triggers the ER stress response (Skowronek et al., 1998). Persistent ER stress induced by viral protein overproduction can lead to the apoptotic death of infected cells (Choi et al., 2019). We found that ER stress caused by HBV proteins upregulates miR-3145-3p expression, which in turn exerts anti-HBV effects.

## Materials and methods

### Cell lines and viruses

HepG2/NTCP (Nishitsuji et al., 2015) and HBV positive HB611 cell lines (Tsurimoto et al., 1987) were cultured in the DMEM/Ham’s F-12 (FHUJIFILM Wako, Osaka, Japan) supplemented with 10% fetal bovine serum (FBS) (Thermo Fisher Scientific, Waltham, MA), 1% non-essential amino acids (Thermo Fisher), 100 unit/mL penicillin, 100 μg/mL streptomycin (Nacalai), and 500 μg/mL G-418 (Promega, Madison, WI). Primary human hepatocytes were purchased from Corning. The cell lines were grown in collagen type I-coated culture dishes (AGC Techno Glass, Shizukoka, Japan) at 37°C in a 5% CO_2_ incubator. HEK293 cells were obtained from American Type Culture Collection (ATCC, Manassas, VA). HEK293T cells were obtained from Horizon Discovery (Waterbeach, United Kingdom). The cells were cultured in RPMI-1640 medium (Sigma-Aldrich, St. Louis, MO) supplemented with 10% FBS, 100 U/mL penicillin, and 100 μg/mL streptomycin at 37°C in a 5% CO_2_ incubator.

To prepare a secreted NanoLuc (secNL) gene harboring HBV (secNL-HBV), pUC1.2xHBV/secNL and pUC 1.2xHBV/D were transfected into HepG2/NTCP cells, and the HBV purification step was performed as previously described (Nishitsuji et al., 2018). HBV from HB611 was also isolated using the same technique used to infect the primary hepatocytes. To prepare miRNA-targeted site-mutated secNL-HBV (secNL-HBVm), primers were designed based on the sequence MH580652.1. SecNL-HBV was used as a template for site-directed mutagenesis using the KOD One (TOYOBO, Osaka, Japan) and specific primers (Table 1).

**Table 1:** Primer sequences for RT-qPCR.

Gene name	Forward primer (5′ → 3′)	Reverse primer (5′ → 3′)
Pri-miR-3145	ACCACTAACTCCATGCAAACT	TGGGAAGTTGACTGTATCGG
POL region	AGGCTTTCACTTTCTCGCCA	GGTTGCGTCAGCAAACACTT
NHSL1	GCAGTTCTCAGTACTACTCTCAG	GAGAAGTCACTTGAGGCTGG
ISG15	GAGAGGCAGCGAACTCATCT	CTTCAGCTCTGACACCGACA
5.8S	GTCTACGCCATACCCT	AAAGCCTACAGCACCCGTA
miR-122-5p	TGGAGTGTGACAATGGTGTTTG	mRQ3 primer
miR-199a-3p	ACAGTAGTCTGCACATTGGTTA	mRQ3 primer
miR-205-5p	TCCTTCATTCCACCGGAGTCTG	mRQ3 primer
miR-210-3p	CTGTGCGTGTGACAGCGGCTGA	mRQ3 primer
miR-3145-5p	AACTCCAAACTCAAAACTCA	mRQ3 primer
miR-3145-3p	AGATATTGAGTGTTTGGAATTG	mRQ3 primer

### Infection of secNL-HBV

HepG2/NTCP cells were infected with secNL-HBV at a multiplicity of infection (MOI) of 10 for 24 h, as previously described (Nishitsuji et al., 2018). On days 3, 6, 9, and 12, the culture supernatants were subjected to a NanoLuc Luciferase Assay Kit (Promega) using the GloMax Navigator System (Promega). Similar experiments were performed using secNL-HBVm.

### Reverse transcription-quantitative polymerase chain reaction

Total RNA was extracted using the ISOGEN (Nippon Gene, Tokyo, Japan). complementary DNA (cDNA) synthesis and reverse transcription-quantitative polymerase chain reaction (RT-qPCR) were performed as previously described (Fekadu et al., 2021) using specific primer sets (Table 2). Briefly, the amplification of the target genes was repeated for 40 cycles with a program of 2 min at 98°C, 10 s at 95°C, and 30 s at 60°C. The expression levels of 5.8S or U6 were used as internal standards. Small RNA cDNA was synthesized using the Mir-X miRNA First-Strand Synthesis Kit (TaKaRa Bio, Shiga, Japan), and 5.8S rRNA or U6 snRNA was used as an internal standard.

**Table 2:** Primer sequences for vector construction.

	Forward primer (5′ → 3′)	Reverse primer (5′ → 3′)
F1	GTACAAGTAACTCGAGCTGACTCAGCATGTCAACGAC	TTGCGCCAGCGCCGCTGTAAGGCCACATGTTGACATCTATAATGTC
F2	GTACAAGTAACTCGAGCTGTTTGA GTTGATCCGCCACT	TTGCGGCCAGCGGCCGCTCCTACCTGATTTGCCTCTCTCTGGCCAATGAT
F3	TACAAGTAACTCGAGGGCATTCTATAAGAGAGAAACTACGCAGTGC	TTGCGGACCAGCGGCCGCAGGAATCGTGCAGGTCTTGCAT
F4	GTACAAGTAACTCGAGGGCCTATACCTTCCTGCTGGTGG	TTGCGGCCGCTGACATACTTTCCAATCAATAGGTCTTACAGGCAG
F5	GTACAAGTAACTCGAGGCCAAGTCTGTACAACATCTTGAGTCCCTTTTTAC	TTGCGGCCAGCGGCCGCAAATGAGGTGTATTTCCGAGAGAGGACAACAGAGTT
F6	GTACAAGTAACTCGAGCGACAACTCTGTTGTCCTCTCTCGGAAATA	TTGCGGCCAGCGGCCGCCCGATACAGAGCTGAGGCGGTG
F5-S1	ATGTTGGGGTACTTTACCGC	CTCGAGGCGATCGCCTAGAATTACTG
F5-S2	ACACAATGTGGCTATCCTGC	CTCGAGGCGATCGCCTAGAATTACTG
F5-S3	GCCAAGTGTTTGCTGACGCA	CTCGAGGCGATCGCCTAGAATTACTG
F5Δ	AACCTTTACCCCGTTGCCCGGCA	AAGTTGGCGAGAAAGTGAAAGCCTGC
F5m	GGCAACGGGGTAAAGGTTCAGAATTCGTTTACACAGA	CAACTTACAAGGCCTTTCTGTGTAAACGAATTCTGAACC
HBS	AAGCTTGGAGGTTGGTCTTCCAAACCTCGAC	GAATTCTCAAATGTATACCCAAAGACAAAAGAAAATTGGTAATAGAGGTAAAAAGGGAC
HBX	AAGCTTGCTGCTAGGGTGTGCTGCCAAC	GAATTCTTAGGCAGAGGTGAAAAAGTTGCATGGTGCTG
BiP	TAGAAGCTTATGAAGCTCTCCCTGGTG	TAGTCTAGACTACAACTCATCTTTTTCTGCTGTATCCTCTTCACCAGTTG

### Construction of HBV proteins and BiP expression vectors

Fragments of the HBV genes and BiPs were amplified using KOD One and specific primers (Table 1). pUC1.2HBV/D and HepG2/NTCP cDNA were used as PCR templates. PCR products were incorporated into pCMV-3XFLAG (Mock, provided by Dr. Koji Nakagawa, Health Science University of Hokkaido School of Nursing & Social Services). The vector was constructed as previously described (Sakata et al., 2020). HepG2/NTCP cells were seeded at 5 × 10^5^ cells/well in a six-well plate, and the expression vector was transfected using Lipofectamine 3,000. RNAs and proteins were collected 48 h later.

### Treatment of cells with IFN-β1 or ER stress inducer HA15

HepG2/NTCP cells were cultured at a density of 1 × 10^6^ cells/well in six-well plates. Recombinant human IFN-β1 (R&D Systems, Minneapolis, MN) was added at a concentration of 100 U/mL, and the cells were incubated for 6 h, or HA15 (MedChemExpress, Monmouth Junction, NJ), which inhibits the ATPase activity of BiPs (Cerezo et al., 2016), was applied at concentrations of 5 μM and 10 μM for 4 h. The total RNA was extracted. The action of IFN-β1 was confirmed by an increase in the expression of IFN-stimulated gene 15 (*ISG15*).

### Establishment of doxycycline-inducible pri-miR-3145-expressing cell lines

Genomic DNA (gDNA) was purified from HepG2/NTCP cells using a GenElute Mammalian Genomic DNA Miniprep Kit (Sigma-Aldrich) and was applied for PCR amplification for pri-miR-3145 using KOD One and specific primers (Table 1). The PCR product was digested with *Xho*I and *Eco*RI and ligated into the pTRIPZ vector (Horizon). The pTRIPZ or the pTRIPZ vector with pri-miR-3145 was inserted, and a translentiviral packaging mix (Horizon) was introduced into HEK293T cells using the TransIT-Lentiviral Transfection Reagent (TaKaRa). HEK293, HepG2/NTCP, and HB611 cells were infected with culture supernatants at an MOI of 10. Cells were cultured in medium containing puromycin and treated with 1 to 10 μg/mL doxycycline (Dox) (TaKaRa) to induce the expression of pri-miR-3145. The Dox-induced expression of 10 nM miRNA was used in the experiments.

### Cell proliferation assay

Cells were seeded at 6.25 × 10^3^ cells/well in a type I collagen coated 96-well plate, and the next day, 1 and 10 10 μg/mL Dox was added. After 48 h, 10 μL of CCK-8 (DOIJNDO, Kumamoto, Japan) was added to 100 μL of culture medium, and the absorbance at 450 nm was measured using a DTX880 (Beckman Coulter, Brea, CA). Cell viability was calculated as 100% of the absorbance of cells treated with medium alone.

### miRNA reporter assay

Primers for cloning the HBV genome fragments were designed such that the full-length viral genome could be divided into six parts. The base sequences of the PCR products with some overlaps were cloned. These HBV genome fragments were amplified using KOD One and specific primers (Table 1) using pUC1.2xHBV/D as a template. Amplified PCR products were inserted into *Xho*I-*Not*I-digested psiCHECK2 using the In-Fusion Snap Assembly Master Mix (TakaRa). The miR-3145-3p target sites in the HBV genome were confirmed by constructing an additional miRNA reporter. Inverse PCR was performed using the F5 fragment of the HBV genome as a template with KOD One; the specific primers are listed in Table 1.

HEK293 cells expressing Dox-induced pri-miR-3145 were seeded at a density of 1 × 10^5^ cells/well (24 wells). Next, 10 ng of the miRNA reporter vector was introduced using Lipofectamine 2000. After 48 h, a Dual-Glo Luciferase Assay Kit (Promega) and GloMax were used to measure luciferase activity. *Renilla* luciferase activity was used to normalize the reporter activity.

### Prediction of miR-3145 target sites in the HBV genome

To confirm the targeting of miR-3145 by the HBV genome, we used an RNA hybrid^1^ based on the principle of miRNA target recognition (Rehmsmeier et al., 2004). Sequences that matched completely with the 5′ seed and partially with the 3′ regions in miR-3145 were selected for the hybridization of the miRNA with potential target viral RNAs. Sequences with a G:U wobble-based pair in their seed sequences were excluded.

### Immunoblotting

Protein samples (5 μg) were electrophoresed using 15% SDS-PAGE, then transferred to a PVDF membrane (Merk Millipore, Brea, CA). The PVDF membrane was treated with Block ACE (KAC, Amagasaki, Japan) to prevent non-specific antibody binding. Subsequently, Specific antibodies against HBS (Mono-1, Beacle, Kyoto, Japan), HBX (X36C, Abcam, Cambridge, United Kingdom), core (10E11, Merk Millipore, Brea, CA), ATF6 (W17028A, BioLegend), C/EBP homologous protein (CHOP) (9C8/CHOP; BioLegend, San Diego, CA), BiP (C50B12; Cell Signaling Technology, Danvers, MA), ATF4 (60035-1-Ig, Proteintech, Rosemont, IL) and glyceraldehyde triphosphate dehydrogenase (GAPDH) (EPR16891; Abcam) were incubated with the PVDF membrane. After washing, the membrane was incubated with horseradish peroxidase (HRP)-conjugated anti-rat IgG (Proteintech, Chicago, IL) or HRP-conjugated anti-rabbit IgG (CST) or HRP-conjugated anti-mouse IgG (CST). Specific antibody-antigen reactions were visualized using Immobilon (Merk Millipore) and detected with X-ray film. GAPDH expression levels were used as an internal standard.

### Introduction of small interfering RNAs or miRNA inhibitors

Two small interfering RNAs (siRNAs) with different sequences targeting *NHSL1* (hs. RiNHSL1.13.2 and hs. RiNHSL1.13.3) or negative control 1 (QIAGEN, Hilden, Germany) were transfected into HB611 cells using Lipofectamine RNAiMAX (Thermo Fisher) according to the reverse-transfection protocol. siRNA was introduced into the cells at a final concentration of 5 nM, and the total RNA was extracted with ISOGEN after 48 h. Similarly, 20 nM miR-3145-3p inhibitor (miRCURY miRNA inhibitor Y104 105715-ACA; QIAGEN) and negative control A (QIAGEN) were introduced into secNL-HBV-infected HepG2/NTCP cells.

### Data analysis of NHSL1 expression levels and pathogenetic relevance for patients with HBV-related disease

The mRNA profiles of patient data for HBV-positive acute hepatitis were analyzed using the GSE38941 dataset from the National Center for Biotechnology Information (NCBI) database. HCC patient data were analyzed using Kaplan–Meier Plotter RNA-seq (Lanczky and Gyorffy, 2021).^2^

### Statistical analysis

The Mann–Whitney *U* test was performed to analyze differences between two independent groups. Data are presented as the mean ± standard deviation. Statistical significance was set at *p* < 0.05.

## Results

### Expression of miR-3145 induced by HBV infection suppresses viral replication

**Figure 1:**
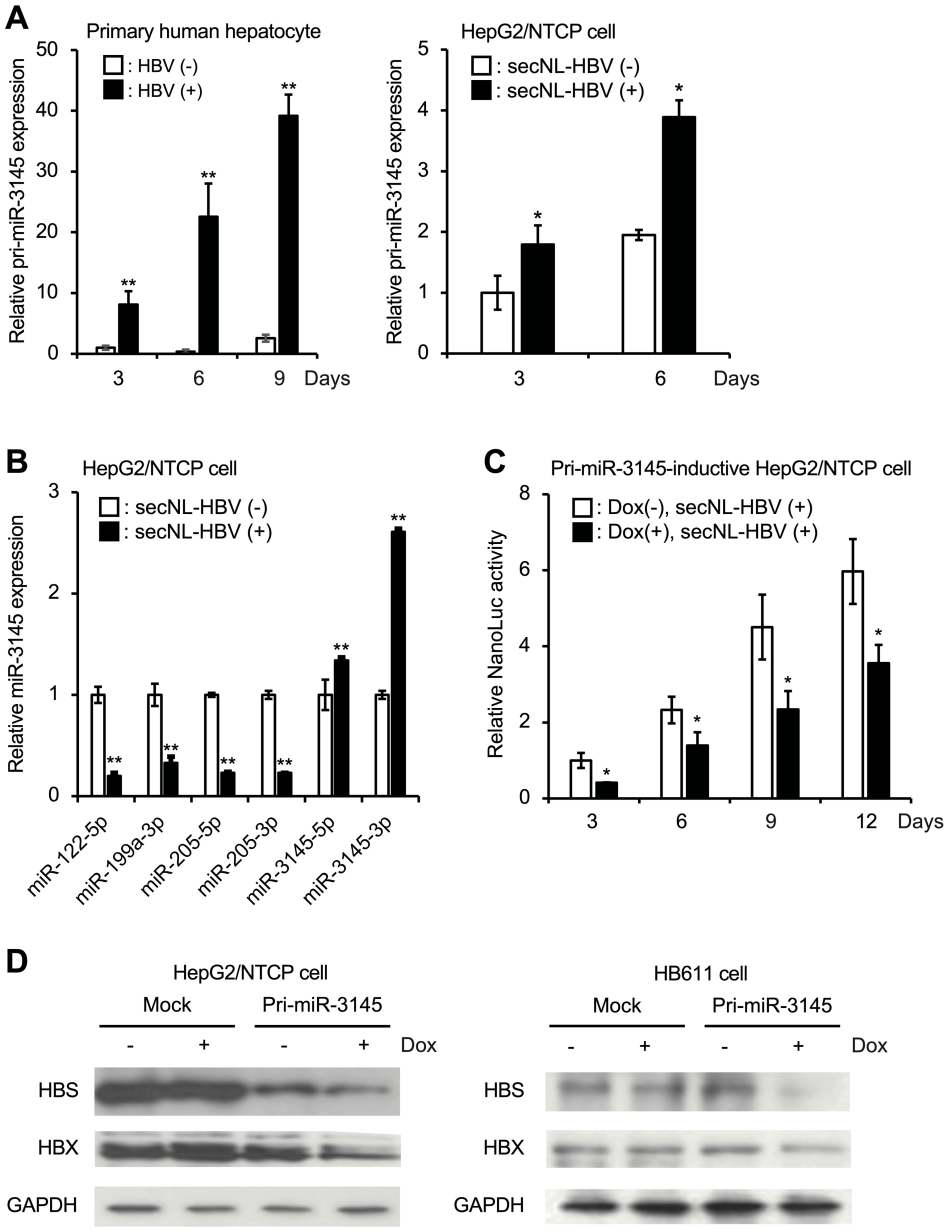
miR-3145, whose expression increases in cells upon hepatitis B virus (HBV) infection, suppresses viral replication. **(A)** Expression of pri-miR-3145 in HBV-infected primary hepatocytes (left) and secreted NanoLuc (secNL)-HBV-infected HepG2/NTCP cells (right). The expression level of pri-miR-3145 was corrected with the expression level of 5.8S ribosomal RNA. **(B)** miR-122-5p, miR-199a-3p, miR-205-5p, miR-210-3p, miR-3145-5p, and miR-3145-3p expressions in secNL-HBV-infected or secNL-HBV-uninfected HepG2/NTCP cells. Each expression level of miRNA was quantified using a reverse-transcription quantitative polymerase chain reaction corrected with the expression level of U6 small nuclear RNA. As controls, the expression level of miRNAs for uninfected cells was set to 1. **(C)** Inhibition of secNL-HBV propagation in HepG2/NTCP cells by a doxycycline (Dox)-induced expression of pri-miR-3145. As a control, NanoLuc (NL) activity on day 3 of Dox-untreated cells was set to 1. **(D)** Suppression of viral HBV S (HBS) and HBV X (HBX) expressions in cells with induced miR-3145 expression. Glyceraldehyde triphosphate dehydrogenase (GAPDH) was used as an internal control. **(Left)** NTCP receptor expressing HepG2 cells. **(Right)** HB611 cells. ^*^p < 0.05 and ^**^p < 0.01.

After primary hepatocytes were infected with HBV, the expression of pri-miR3145 was upregulated over the course of 1 day (Figure 1A, left). This phenomenon was also observed when a recombinant HBV possessing secNL was infected with HepG2/NTCP cells (Figure 1A, right) (Nishitsuji et al., 2015; Tsurimoto et al., 1987). Several host miRNAs such as miR-122 have been reported to suppress HBV infection (Lamontagne et al., 2015; Liu et al., 2019). However, the expressions of miR-122, miR-199, miR-205, and miR-210 were significantly decreased in secNL-HBV-infected HepG2/NTCP cells compared to those in uninfected cells (Figure 1B). In contrast, secNL-HBV infection increased miR-3145-5p and miR-3145-3p expression 1.4-fold and 2.5-fold, respectively (Figure 1B).

To clarify the role of miR-3145 in HBV infection, HepG2/NTCP cells expressing pri-miR-3145 in a Dox-dependent manner were generated and infected with sec-NL-HBV in the presence of 1 μg/mL Dox. The activity of NL secreted into the culture supernatant was proportional to the number of HBV genomes amplified in the cells (Nishitsuji et al., 2015). Overexpression of pri-miR-3145, with the addition of Dox, suppressed NL production on days 3, 6, 9, and 12 (Figure 1C). Furthermore, HepG2/NTCP cells overexpressing pri-miR-3145 were infected with secNL-HBV, and only cells expressing pri-miR-3145 showed reduced HBS and HBX expressions (Figure 1D, left). Similarly, the overexpression of pri-miR-3145 in HB611 cells continually infected with HBV (Tsurimoto et al., 1987) was linked to the reduced expressions of HBS and HBX (Figure 1D, right). These results indicated that miR-3145, whose expression is upregulated by HBV infection, suppresses HBV replication.

### miR-3145-3p binds the HBV genome sequence in the pol region and suppresses HBV replication

**Figure 2:**
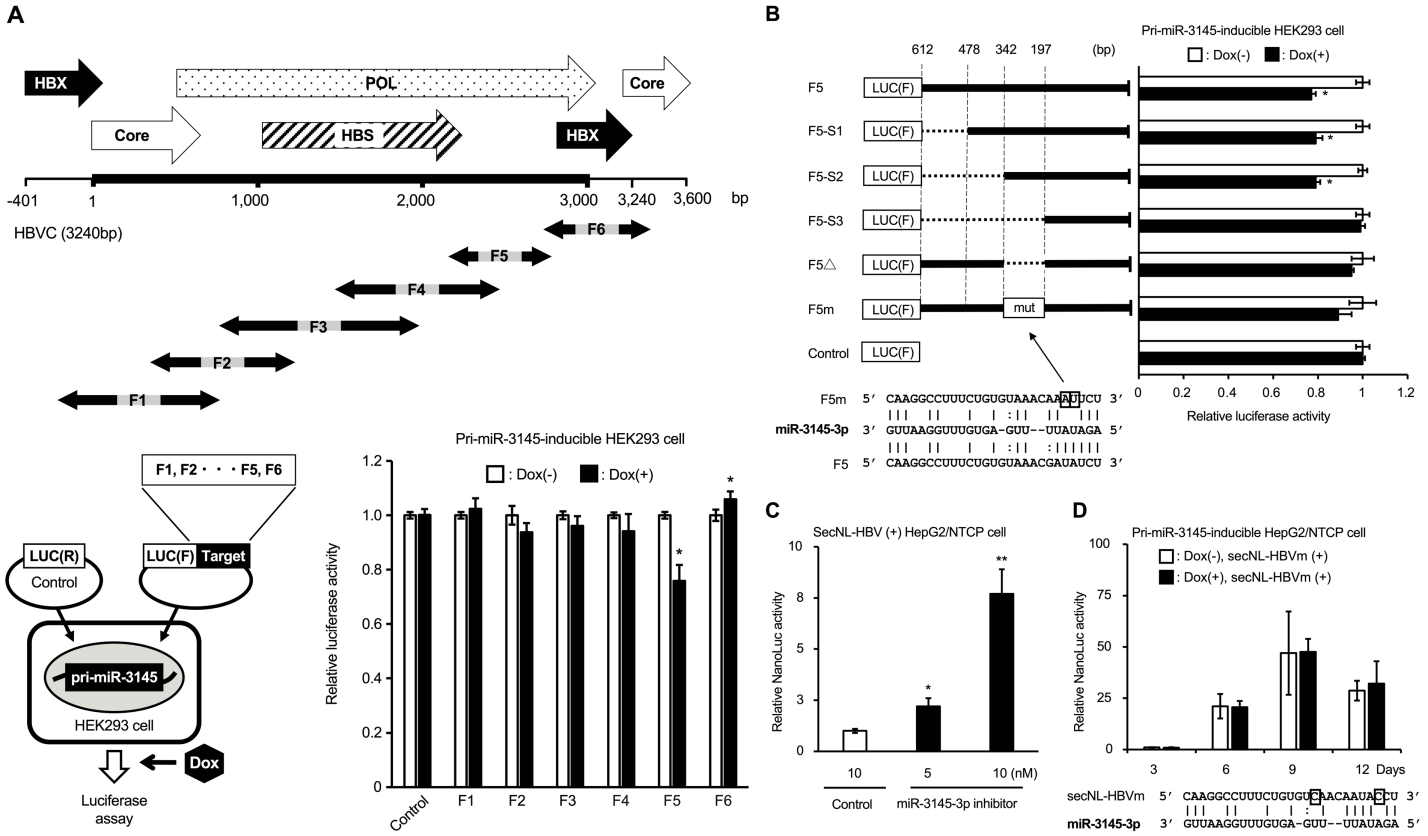
miR-3145-3p targets the pol region of the hepatitis B virus (HBV) genome. **(A)** Identification of miR-3145 target sites on the HBV genome sequence by the luciferase assay. **(Upper)** Overlapping partial HBV genome sequences from F1 to F6 were constructed to encompass a 1.2-fold length of the entire HBV genome sequence. **(Lower left)** HepG2/NTCP cells that express pri-miR-3145 in a doxycycline (Dox)-dependent manner were transfected with a plasmid in which one of the HBV gene regions from F1 to F6 was inserted downstream of the Firefly luciferase [Luc (F)] gene together with a plasmid expressing *Renilla* luciferase [Luc (R)]. **(Lower right)** Relative Firefly luciferase activity compared to the *Renilla* luciferase activity was investigated in miR-3145-inducible HepG2/NTCP cells transfected into each HBV gene region. The value for Dox-untreated cells was set to 1. **(B)** Identification of miR-3145 target sites by transfecting each plasmid containing an HBV gene with different deletions or mutations in pri-miR-3145-HEK293 cells. F5-S1: del478-612; F5-S2: del342-612, F5-S3: del197-612; F5Δ: del197-342 that lacks the miR-31545-3p target site; F5m: the target site of miR-3145-3p was disrupted by nucleotide substitutions. As controls, the value for Dox-untreated cells was set to 1. **(C)** Relative NanoLuc (NL) activity in secreted NanoLuc (secNL)-HBV-infected HepG2/NTCP cells treated with miR-3145-3p inhibitor. The NL activity of 10 nM of control inhibitor-treated cells shown in the white bar was set to 1. Black bars indicate NL activity when secNL-HBV infected cells were treated with 5 nM (left) and 10 nM (right) of miR-3145-3p inhibitor, respectively. **(D)** NL activities of HepG2/NTCP cells infected with secNL-HBV with mutations at the miR-3145-3p target site. White bars indicate NL activities of HepG2/NTCP cells infected with secNL-HBV with two mutations at the miR-3145-3p target site after 3, 6, 9, and 12 days of cultivation, respectively. Black bars indicate NL activities upon pri-miR-3145 overexpression by 3, 6, 9, and 12 days of Dox treatment, respectively. NL activity of Dox-free HepG2/NTCP cells infected with mutated secNL-HBV on day 3 was set to 1. ^*^p < 0.05 and ^**^p < 0.01.

The HBV genome sequence, which was 1.2-fold longer because of partial duplication in the HBX and core regions, was split into six fragments (F1–F6). To confirm whether miR-3145 inhibits HBV gene expression by direct binding, each fragment of the HBV gene was inserted downstream of the luciferase gene into the expression plasmid (Figure 2A). These plasmids were transfected into HEK293 cells to induce pri-miR-3145 expression using Dox, and the luciferase expression was measured. Only the region downstream of pol (F5) showed a 24% reduction in luciferase activity upon overexpression of miR-3145 (Figure 2A). This region was examined using RNA-hybrid, a software program that predicts miRNA-mRNA binding, and a sequence that binds to miR-3145-3p was identified (Figure 2B). miR-3145-3p binds to an enhancer I sequence (342–197 bp in F5), which is important for HBV replication (Figure 2B) (Guo et al., 1991). The luciferase activities in cells transfected with the vector lacking the miR-3145-3p binding sequence (F5Δ) or the vector with a mutation in the binding site of the miR-3145-3p seed sequence (F5m) were not reduced by the pri-miR-3145 expression (Figure 2B).

Experiments were performed to confirm that miR-3145-3p inhibited HBV infection. When secNL-HBV-infected HepG2/NTCP cells were treated with the miR-3145-3p inhibitor, NL activity in the culture supernatants increased according to the amount of miR-3145-3p inhibitor (Figure 2C). Furthermore, similar to control miRNA, HBV infection with a mutation in the miR-3145-3p target site (secNL-HBVm) was not inhibited by pri-miR-3145 (Figure 2D). Moreover, NL production on day 9 after viral infection was approximately 50-fold higher in secNL-HBVm-infected cells that did not express pri-mir-3145 than in wild-type virus-infected cells (Figure 2D). In addition, forced expression of miR-3145 precursor did not affect cell proliferation (Supplementary Figure S1). These results suggested that miR-3145-3p targets the *pol* gene region of the HBV genome and suppresses HBV replication.

### ER stress induces miR-3145 expression

**Figure 3:**
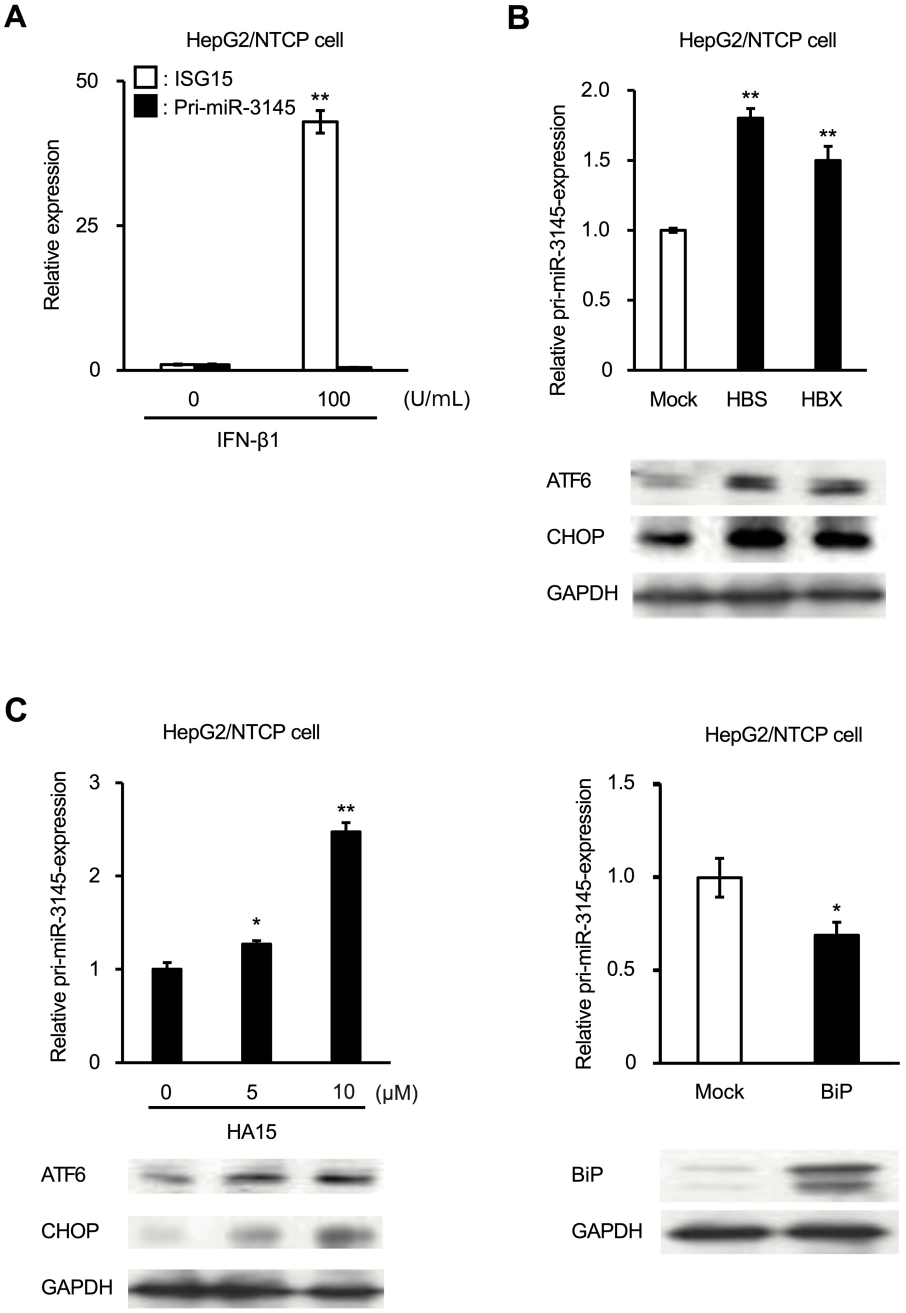
Expression of pri-miR-3145 is induced by endoplasmic reticulum (ER) stress. **(A)** Treatment of HepG2/NTCP cells with 100 U/mL of interferon (IFN)-β1. The white and black bars indicate the relative expressions of interferon (IFN)-stimulated gene 15 (*ISG15*) and pri-miR-3145, respectively. Expression of either ISG5 or pri-miR-3145 without IFN-β1 treatment was defined as 1. **(B)** Pri-miR-3145 is expressed in HepG2/NTCP cells in association with the expression of hepatitis B virus (HBV) structural proteins HBV S (HBS) or HBV X (HBX). The white bar indicates the expression level by the introduction of an empty vector (Mock), which was defined as 1. The black bars indicate the relative expressions by the introduction of HBS and HBX, respectively. **(C)** Relative expressions of pri-miR-3145. **(Left)** Induction of pri-miR-3145 in cells treated with ER stress inducer HA15. HepG2/NTCP cells were treated with HA15 at concentrations of 0, 5, and 10 μM, and the expression level of pri-miR-3145 was quantified by a quantitative polymerase chain reaction (qPCR) after 4 h. The expression level of 0 μM HA15-treated cells was set to 1. **(Right)** Suppression of pri-miR-3145 in HepG2/NTCP cells transfected with a vector that express binding immunoglobulin protein (BiP), a suppressor of ER stress. Cells were transfected with 2.5 μg of either BiP or empty vector plasmid DNA. The expression level of pri-miR-3145 was corrected using 5.8S ribosomal RNA as an internal standard, and the expression level of cells transfected with an empty vector was set to 1. BiP expression was also confirmed by immunoblotting. Glyceraldehyde triphosphate dehydrogenase (GAPDH) was used as an internal standard for protein expression levels. ^*^p < 0.05.

We investigated the molecular mechanism by which HBV infection induces the expression of pri-miR-3145. First, we hypothesized that the innate immune response induced by HBV infection induces the expression of pri-miR-3145. Therefore, we treated HepG2/NTCP cells with IFN-β1. The results showed a 43-fold increase in the expression of *ISG15*, a gene downstream of type I IFN; however, there was no increase in pri-miR-3145 expression (Figure 3A). Viral HBS and HBX proteins are known to activate ER stress (Cho et al., 2011). Overexpression of HBS and HBX in HepG2/NTCP cells increased pri-miR-3145 expression by 1.8-fold and 1.5-fold, respectively (Figure 3B). Next, treatment of HepG2/NTCP cells with 5 and 10 μM of HA15, an inducer of ER stress by specifically targeting BiP (Cerezo et al., 2016), showed a dose-dependent increase in the pri-miR-3145 expression (Figure 3C, left). Furthermore, the expression pattern of pri-miR-3145 correlated with the expression levels of the ER stress markers ATF4, ATF6, and CHOP, which are transcription factors that initiate cell apoptosis (Figures 3B,C, lower; Supplementary Figure S2). However, the expression of pri-miR-3145 was significantly reduced by 31% in the HepG2/NTCP cells transfected with 2.5 μg of plasmid that expresses BiP, a suppressor of ER stress (Cerezo et al., 2016) (Figure 3C, right). The upregulation of BiP by transfection with the BiP-expressing plasmid was confirmed by immunoblotting (Figure 3C, right lower panel). These results indicated that the ER stress response associated with viral infection induces miR-3145 expression.

### Expression of the *NHSL1* gene that encodes pri-miR-3145 indicates a favorable prognosis for virus-positive HCC

**Figure 4:**
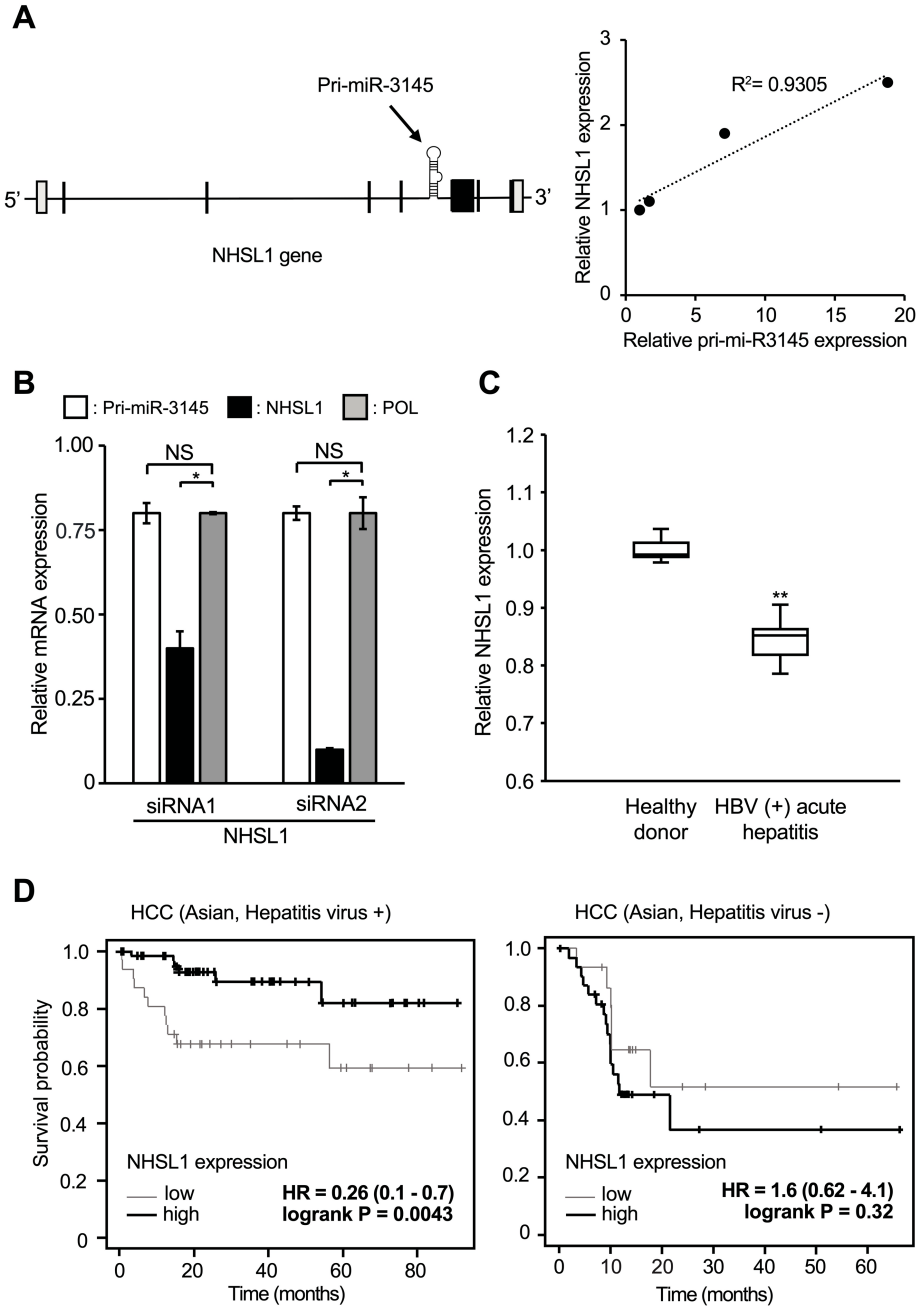
Pri-miR-3145 is encoded in the intronic region of the *NHSL1* gene, which is a good prognostic factor for patients with hepatitis virus-associated hepatocellular carcinoma (HCC). **(A)** *NHSL1* encoding pri-miR-3145 in its intronic region is induced by hepatitis B virus (HBV) infection. **(Left)** Structure of the *NHSL1* gene containing pri-miR-3145. **(Right)** Expression levels of *NHSL1* and pri-miR-3145 are correlated in HBV-infected cells. *Y*-axis, expression level of the *NHSL1* gene. *X*-axis, expression level of pri-miR-3145. Samples were collected on days 3, 5, 7, and 9 after infection of HepG2/NTCP cells with secreted NanoLuc (secNL)-HBV. Gene expression levels were corrected for 5.8S ribosomal RNA expression. **(B)** Expression levels of *NHSL1*, pri-miR-3145, and HBV *pol* genes in HBV-positive HB611 cells treated with *NHSL1* siRNA1 or siRNA2. The expression level of each gene was corrected with the expression level of 5.8S ribosomal RNA. The expression level of cells introduced with the control siRNA was set to 1 (not indicated). NS: no significant difference. **(C)** Box-and-whisker plots (median; box, 25–75%; whisker, minimal/maximal data) of *NHSL1* gene expression levels compared between healthy participants and HBV-positive acute hepatitis patients. The vertical axis indicates relative *NHSL1* mRNA expressions among the total number of reads by next-generation sequencing. **(D)** Time-course changes in the survival rate caused by differences in *NHSL1* gene expression levels in hepatitis virus-associated HCC patients **(left)** and hepatitis virus-negative HCC patients **(right)** in Asian populations using the Kaplan–Meier plotter. ^*^p < 0.05 and ^**^p < 0.01.

Pri-miR-3145 is encoded by an intron of the *NHSL1* gene (Figure 4A, left). The expression levels of pri-miR-3145 and *NHSL1* during HBV infection were quantified using RT-qPCR. The results showed that the expressions of pri-miR-3145 and *NHSL1* were positively correlated (Figure 4A, right), which is consistent with the fact that *NHSL1* and pri-miR-3145 are regulated by a single promoter.

miR-3145-3p targets HBV; however, the role of the *NHSL1* gene in viral infection remains unclear. Therefore, HB611 cells persistently infected with HBV were treated with two siRNAs targeting *NHSL1*. Because mRNAs are spliced in the nucleus and then translocated to the cytoplasm, even a small amount of siRNA can destroy mature mRNA (Bartel, 2009). The expression of *NHSL1* was significantly reduced by 60 and 90% with siRNA1 and siRNA2, respectively, compared to controls (Figure 4B). However, when HB611 cells were treated with *NHSL1* siRNA1, the expression of both pri-miR-3145 and the pol region of HBV was reduced by only 20% compared with the controls (Figure 4B).

Thus, cells with high *NHSL1* expression also highly expressed miR-3145-3p. Because miR-3145 has been identified only recently, clinical data of miR-3145 on the Internet are limited. However, more clinical data of the expression level of *NHSL1* are available from HBV patient datasets. Therefore, we examined the HBV-derived acute hepatitis patient data from the NCBI database. We found that *NHSL1* mRNA expression was decreased in the hepatocytes of acutely infected patients compared to healthy controls (Figure 4C). Because most HCC patients in Asian populations are infected with HBV (Cancer Genome Atlas Research Network, 2017), we used the Kaplan–Meier plotter (Lanczky and Gyorffy, 2021) to examine the correlation between *NHSL1* expression levels and the prognosis of HCC patients in an Asian population. For patients with virus-positive HCC, higher *NHSL1* mRNA expressions were associated with a significantly better prognosis, with a hazard ratio (HR) of 0.26 (range, 0.1–0.7; *p* = 0.0043) (Figure 4D, left). In contrast, for patients with virus-negative HCC, the HR of *NHSL1* mRNA expression and the prognosis was 1.6 (range, 0.62–4.1), with no correlation (*p* = 0.32) (Figure 4D, right). These results strongly suggest that pri-miR-3145 suppresses HBV infection, and that its expression level may be associated with the prognosis for virus-positive HCC patients.

## Discussion

**Figure 5:**
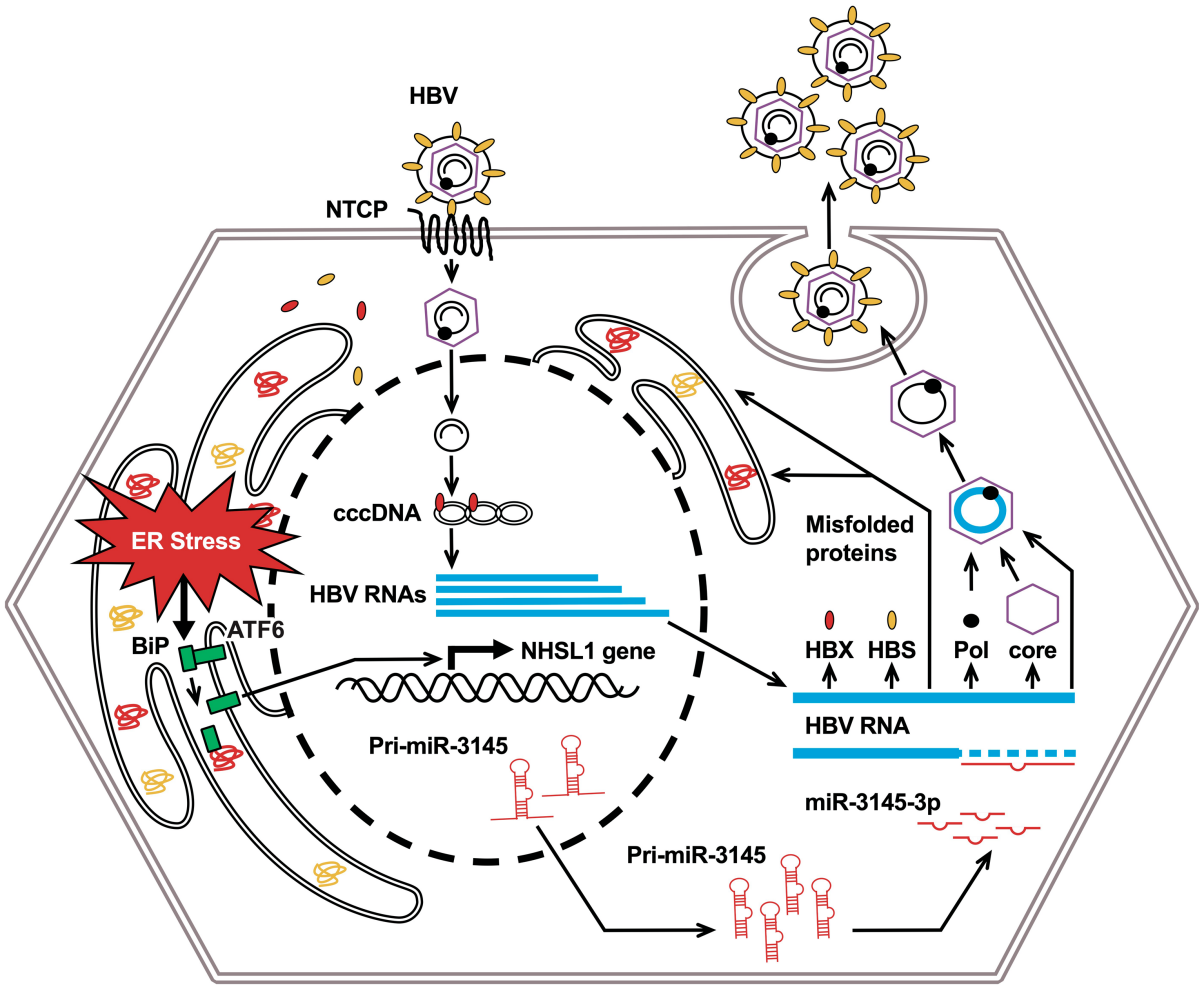
Conceptual diagram of this research: miR-3145 induced by endoplasmic reticulum (ER) stress associated with hepatitis B virus (HBV) infection suppresses viral replication.

When the virus infects cells and excess viral proteins accumulate in the ER, BiPs dissociate from the sensor proteins, which is called ER stress. ER stress induces the phosphorylation of protein kinase R and the downstream expression of CHOP and DNA damage-inducible protein 34, leading to apoptosis of virus-infected cells (He, 2006). For example, dengue virus infection reduces BiP expression and leads to the accumulation of sensor proteins in cells, causing ER stress and reducing viral production (Wati et al., 2009). We showed that the novel miR-3145-3p, whose expression was induced by ER stress, exhibited anti-HBV activity (Figure 5).

Pri-miR-3145 is encoded in the intron region of *NHSL1*, and its expression level correlated with that of NHSL1 (Figure 4A, right). NHSL1 is a tumor suppressor that negatively regulates lamellipodia stability and, thus, cell migration efficiency (Law et al., 2021). In an Asian population that was predominantly HBV-positive, HCC patients with high *NHSL1* expression had better prognoses than those with low *NHSL1* expression (Figure 4D, left). In contrast, in the virus-negative population, high *NHSL1* expression was not a favorable prognostic factor for HCC (Figure 4D, right panel). These results suggest that miR-3145-3p is a favorable prognostic factor for patients with HBV-positive HCC.

Ninety-seven percent of persistent HBV infections worldwide are caused by HBV genotypes A–E (Velkov et al., 2018). The seed sequence binding site of miR-3145-3p on the HBV gene is highly conserved in genotype C. Additionally, the miR-3145-3p seed sequence-binding site was frequently found in genotype B and in 20% of genotypes D, but infrequently in genotypes A and E (Hayer et al., 2013). Notably, miR-3145-3p suppresses genotype C proliferation. Genotype C is less responsive to treatment with IFN (Wai et al., 2002) and is more likely to be associated with chronic hepatitis and HCC development (Orito et al., 2001; Kobayashi et al., 2002).

Referring to the nucleic acid sequences of HBV gene products (Yuen, 2021), miR-3145-3p has binding sequences in pgRNA, pcRNA, preS1 mRNA, and preS2/S mRNA, but not in HBX mRNA. However, pri-miR-3145 overexpression in HBV-infected cells decreased HBX expression (Figure 1D). The decreased HBX expression was probably caused by the reduced production of viral proteins in the cells as a result of decreased pgRNA synthesis.

Treatment of infected cells or mouse models with siRNA, which reduces HBV pgRNA, decreases both viral RNA expression and replication (Giladi et al., 2003; Uprichard et al., 2005). Therefore, we believe that induction of miR-3145 expression may serve as a treatment for HBV infection. Nucleic acid modifications, such as N-acetylgalactosamine, and nucleic acid transport technologies, such as anionic lipid nanoparticles, have been developed. Therefore, RNA can be delivered to the liver (Cui et al., 2021; Pattipeiluhu et al., 2022). Such new technologies for the modification and transport of nucleic acids increase the potential for the development of anti-HBV drugs using miR-3145-3p or small molecules that bind to its target sequence of miR-3145-3p.

## Data Availability

The original contributions presented in the study are included in the article/Supplementary material, further inquiries can be directed to the corresponding authors.

## Footnotes

^1^ https://bibiserv.cebitec.uni-biele.org.de/rnahybrid/

^2^ https://kmplot.com/analysis/
