# Supplementary figures and images for: MicroRNA-3145 as a potential therapeutic target for hepatitis B virus: inhibition of viral replication via downregulation of HBS and HBX
Source: Front Microbiol. 2025 Jan 6;15:1499216. doi: 10.3389/fmicb.2024.1499216 (PMC11743939; doi:10.3389/fmicb.2024.1499216)

Relative cell viability

Control  
Pri-miR-3145

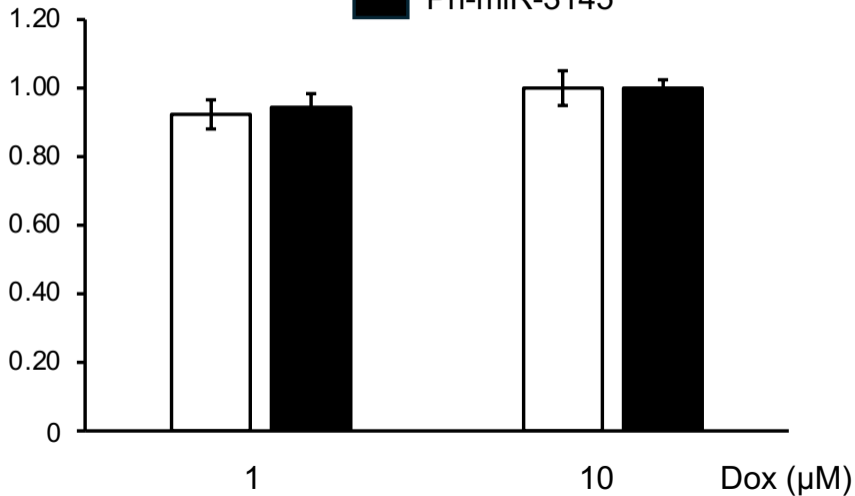

Supplement: Supplementary file 3 [file Data_Sheet_3.pdf]

0 5 10 HA15 ( $\mu$ M)

ATF4

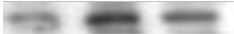

GAPDH

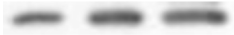

Supplement: Supplementary file 4 [file Data_Sheet_4.pdf]
